# VLM-Nav: Mapless UAV navigation using monocular vision driven by vision-language models

**DOI:** 10.1371/journal.pone.0345778

**Published:** 2026-04-03

**Authors:** Gobinda Chandra Sarker, AKM Azad, Sejuti Rahman, Md Mehedi Hasan

**Affiliations:** 1 Department of Electrical and Computer Engineering, University at Albany, Albany, New York, United States of America; 2 Department of Robotics and Mechatronics Engineering, University of Dhaka, Dhaka, Bangladesh; 3 Department of Mathematics and Statistics, Faculty of Science, Imam Mohammad Ibn Saud Islamic University (IMSIU), Riyadh, Saudi Arabia; 4 Department of Computer Science, New Uzbekistan University, Tashkent, Uzbekistan; National University of Sciences and Technology, PAKISTAN

## Abstract

Autonomous vehicles, such as Unmanned Aerial Vehicles (UAVs), have the potential to completely reshape various industries such as parcel delivery, agriculture, surveillance, monitoring, and search-and-rescue missions. Consequently, the demand for safe, cost-effective, and intelligent navigation systems is crucial to ensure reliable performance in complex and dynamic environments. In this study, we propose a novel vision-based UAV navigation method that integrates depthmap estimation with a Vision-Language Model (VLM) for efficient obstacle avoidance and path planning. The system processes RGB images captured by the UAV, transforming them into depth maps using DepthAnything-V2, a powerful zero-shot depth estimator. These depth maps are then analyzed by the VLM, which detects nearby obstacles and plans avoidance maneuvers. We have explored the Gemini-flash and GPT-4o models as VLM in our study. A fully connected network integrates the VLM output with the UAV’s relative heading angle to predict the optimal course of action, enabling the UAV to dynamically navigate complex environments toward its target. The system’s effectiveness is validated through simulations in AirSim using Blocks and the Downtown West environment. The UAV consistently reaches its destination, avoiding obstacles and achieving a near-perfect task completion rate of 0.98. By eliminating the need for costly sensors such as LiDAR and operating without pre-existing maps, our solution provides a cost-efficient, generalizable approach to real-time UAV navigation, especially in unfamiliar or dynamic settings, and highlights emerging trends in autonomous systems research that utilize VLMs.

## 1 Introduction

Autonomous UAV systems are poised to redefine the future by seamlessly integrating intelligence, adaptability, and efficiency across a wide range of industries. By navigating complex terrains, autonomous UAVs will unlock new possibilities in urban air mobility, disaster relief, and sustainable practices [1–3]. The task of autonomous UAV navigation can be viewed as a process in which the UAV develops a strategy to reach a designated destination safely and efficiently without human intervention. The Key features of such systems include real-time obstacle avoidance, map independence, and adaptability to unknown terrains. These capabilities are achieved by integrating advanced AI and sensor technologies, which enable the seamless processing of complex visual and spatial data, ensuring safe, efficient, and responsive flight paths. To successfully complete its designated mission, a UAV requires comprehensive awareness of its surroundings, including its location, velocity, heading direction, and target destination [4,5]. The UAV first acquires sensor data regarding its current state through the perception module, which is then sent to the planner module for tasks such as path planning, collision avoidance, localization, and the generation of continuous control signals. These control signals guide the UAV toward its desired destination.

The perception module utilizes a wide range of sensors. In comparison to traditional sensors, such as LiDAR and ultrasonic sensors, visual sensors offer distinct advantages, as they can capture detailed information about the surroundings, including color, texture, and other visual details. Additionally, visual sensors are more cost-effective and easier to deploy, making them a focal point of research in the field of navigation [6]. The types of visual sensors typically include monocular, stereo, RGB-D, and fisheye cameras. Monocular cameras, specifically, are well-suited for applications where compactness and minimal weight are crucial factors, and thus they are the primary focus of this research. Table 1 highlights the merits and demerits of monocular sensors compared to LiDAR. Vision-guided navigation systems are categorized into three main groups: map-independent, map-based, and map-building techniques. Map-independent (mapless) systems function without a predefined map; instead, they rely on observing and extracting distinctive environmental features to navigate, as described by [4]. In this study, we focused on developing such a system, as it offers several advantages over map-based alternatives. Notably, it eliminates the need for a global map, which is a requirement in map-based methods [7,8]. Map-building techniques encounter difficulties in highly dynamic environments and face challenges related to the time-consuming creation and updating of obstacle maps [9,10]. Compared to map-based UAV navigation, the mapless approach eliminates the need for complex path planning or path tracking while enhancing the system’s ability to handle dynamic obstacles.

**Table 1:** Comparison between different sensors for perception.

Sensor	Price Range (USD)	Range (Meters)	Weight (kg)	Accuracy (cm)	Power (W)
LiDAR	$1,000 – $75,000	100–300+	0.3–1.5	2–5	5–15
Monocular Camera	$50 – $500	10–50	< 0.1	Varies	1–2
Stereo Camera	$150 – $2,000	10–50	0.1–0.5	5–10	3–5
RGB-D Camera	$150 – $5,000	5–10 (active)	0.2–0.7	1–3	3–10

Over the past few years reinforcement learning (RL) methods became popular for the navigation task [11]. It allows for an end-to-end learning mechanism, where UAV learns to map directly sensory input to control action. Unlike the optical flow or feature tracking methods, it learns from mistakes, which gradually improves navigation performance over time. Furthermore, obtaining labelled training data can be difficult using supervised and motion based approaches. However, many prior researchers utilizing RL for UAV navigation typically train their models in static, predefined environments [12,13]. In such setups, the RL agent learns specific visual and spatial features within the training environment, which limits its generalizability to unseen or more complex settings with different lighting, textures, or obstacle configurations, leading to poor real-world performance [14,15]. Moreover, training RL policy can be computationally expensive and time-consuming. The UAV must interact with the environment repeatedly, collecting experience through trial and error. This requires extensive simulations or real-world flight data, both of which come with high computational overhead. RL algorithms also often involve complex reward structures and tuning, which further increases training time [16].

VLMs and Large Language Models (LLMs) have shown significant success in various tasks, including image-to-text generation, object detection, semantic segmentation, and content creation [17]. These advancements are creating new opportunities for decision-making in navigation, especially within vision-and-language navigation (VLN) [18]. Recently, researchers have been investigating the use of LLMs and VLMs for robotic navigation. These models are particularly advantageous due to their zero-shot learning capabilities and their ability to explain reasoning, enabling effective navigation in unfamiliar settings. Typically, a VLN agent navigates by following a series of human-given instructions and utilizing visual cues to reach a designated target, with success measured by proximity to the goal location [18]. While the integration of VLMs in autonomous vehicles is still being developed, the increasing sophistication of cutting-edge models indicates their potential for broader applications in this area.

**Fig 1:**
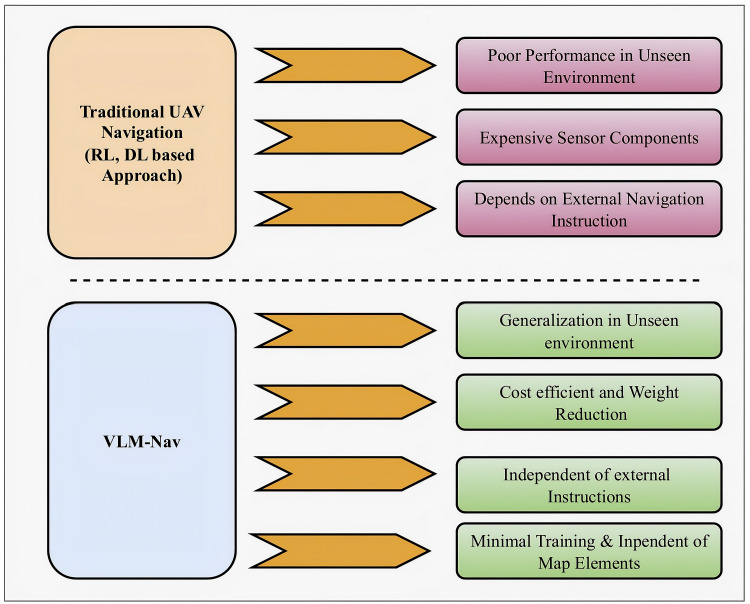
Compared to traditional RL/DL-based UAV navigation systems which struggle with unseen environments, require expensive sensors, and rely on external instructions, VLM-Nav offers better generalization, cost efficiency, independence from external inputs, and minimal training needs, making it a more adaptable and efficient navigation approach.

In this research, we have developed VLM-Nav, a cost-efficient method for controlling a multirotor UAV to travel from its starting point to a designated destination while avoiding collisions with obstacles, utilizing visual data obtained from an onboard monocular camera. Since depth perception cannot be achieved with RGB images alone, the RGB images are transformed into a depth map using a pretrained depth-estimation model, which provides crucial spatial information about the environment. The depth maps, along with specific prompts, are then fed into a VLM, which processes the visual data and generates context-aware decisions related to obstacle avoidance. The outputs from the VLM, along with the heading angle, are input into a fully connected network (FCN). This model predicts the optimal action at each timestep, ensuring that the UAV navigates safely while avoiding obstacles in real-time [19]. Our objective is to replicate human-like decision-making for UAV control. Fig 1 illustrates the advantages of the proposed system compared to previous methods in the literature. Our approach enhances generalizability across varied environments by decoupling perception and decision-making. This contrasts with many prior research approaches that tend to overfit to specific training environments [14,20,21], where UAVs learn environmental features such as color, lighting, and textures that may not be relevant in new, unseen environments. We achieved the following contributions in our work.

- This study develops an end-to-end autonomous UAV navigation framework guided by monocular vision.
- The proposed method relies on RGB scene images, which are converted into depth maps using a zero-shot pretrained depth estimator and heading angle, making it fully map-independent.
- By separating perception from decision-making, the approach enhances generalizability and explainability, allowing the UAV to navigate diverse and complex environments.
- Unlike prior research in visual language navigation in the literature, the proposed method does not rely on any external instructions from humans or utilize any extensive training and dataset.
- This research offers insights into the use of VLMs for UAV navigation and explores current challenges and future research directions.

Section 2 provides a comprehensive overview of previous research on vision-guided navigation systems and discusses the remaining challenges. In Section 3, we outline the specific navigation task this study focuses on. The methodology for our proposed VLM-Nav approach is detailed in Section 4, followed by the simulation results in Section 5. The contributions of each component in our system are provided in section 5.5. Finally, Section 6 summarizes the study’s key findings.

## 2 Literature review

Although UAVs have been utilized in various applications over the years, achieving safe flight during autonomous navigation remains a significant challenge [6]. Map-based UAV navigation relies on pre-defined 2D or 3D environmental representations. These maps vary in complexity, incorporating terrain details, obstacle locations, and strategic waypoints. Cui et al. [7] introduced a multi-layer strategy based on RL algorithm leveraging both global and local informations for efficient path planning. However the study was carried out in a grid-based environment, where UAV movements are defined by eight possible actions within a two-dimensional state space. Kim et al. [22] utilized DL-based object detection to identify and label ground objects, converting them into labeled points. The spatial configuration of these objects is then matched with a pre-existing map database to determine the UAV’s precise location. Other approaches [23,24] leverage GPS signals, georeferenced maps, and optical flow techniques to refine UAV positioning. Additionally, Simultaneous Localization and Mapping (SLAM) has emerged as a key method for real-time map generation, allowing UAVs to navigate without relying on pre-existing maps. SLAM frameworks, such as LIO-SAM [25], integrate LiDAR and inertial data to enhance accuracy, particularly in complex outdoor environments. While map-based strategies offer reliable navigation, their effectiveness depends on the availability and accuracy of pre-existing maps. Consequently, mapless navigation, which leverages onboard sensors and AI-driven adaptability, has attracted attention for its robustness in dynamic or unfamiliar terrain, making it the focus of this research. The various approaches discussed in the literature are systematically compared in Table 2.

**Table 2:** Recent studies in vision based UAV navigation.

Ref	Objective	Method	Action Space	Simulation Setup	Limitations
[21]	Indoor navigation & obstacle avoidance	VAE & SAC (delayed learning)	Continuous Control	Airsim	• Performance decrease in new environment
[26]	Indoor navigation	DDPG	velicity (x,y)	Airsim	• Simple environment. • No target location considered
[12]	Indoor & outdoor navigation	Memory Enhanced DQN	yaw left, yaw right, straight	Airsim	Simulation only, Fixed height
[20]	Obstacle avoidance using monocular vision	FCRN (depth estimation) & D3QN	15 discrete actions (linear & angular velocity)	ROS and Gazebo	• Cannot generalize well in unseen environment.
[27]	Indoor navigation	H-DrQ & temporal attention	Continuous Yaw Angle [−60,60]	Airsim	• Cannot generalize well in unseen environment. • High computational cost.
[28]	3D UAV navigation	DDPG & TD3 with human in the loop	Acceleration ($a_{x},a_{y},a_{z}$)	Airsim	• Not considered collision in the reward function. • Not considered how relative target location will be received
[29]	Visual language navigation	CNN + Bi-LSTM+GRU with cross modal attention	series of action (Move forward, left right, up and down)	Airsim	• Requires external instruction
[30]	Visual language navigation	LLM (GPT-3, GPT-4)	series of action	Airsim	• Requires external instruction. • Need to specify landmarks. • Requires high computational resources.
[31]	MultiObject Navigation	Scene Object Graph + GPT-3.5-turbo/GPT-4 + Imitation Learning	Forward, left, right	–	• Requires high computational resources
**Proposed**	UAV Navigation and obstacle avoidance	DepthAnything + VLM	Forward, Yaw left and Yaw right, Up and Down	Airsim	• Only static obstacles considered.

Early methods for monocular vision-based navigation include appearance-based approaches that treat obstacles as foreground objects against a consistent background, like the ground or sky [32]. These methods are typically limited to environments where obstacles are easily distinguishable from their surroundings. Motion-based techniques, such as optical flow, rely on the assumption that nearby objects show distinct movements detectable through motion vectors in the image. Since objects closer to the camera have greater displacements, any point with a displacement exceeding a specific threshold is classified as an obstacle pixel [33]. However, the effectiveness of optical flow estimation is often poor because it assumes brightness constancy and smooth transitions between frames, conditions that are rarely fulfilled in real-world situations [34]. Another method is feature tracking. For example, the study in [35] uses a monocular camera to estimate the depth of obstacles in both indoor and outdoor environments. The algorithm employs SURF (scale-invariant feature transform) to achieve this. At each step, the depth of an obstacle is estimated using a Kalman filter. When the estimated distance to an obstacle exceeds a certain threshold, a hover command is triggered, prompting the UAV to rotate before continuing forward.

RL-based methods are widely used for robotic navigation tasks because they effectively overcome the limitations of earlier techniques. Chen et al. [13] introduced a method for collision-free UAV navigation in small indoor environments using monocular camera images. The proposed technique utilizes object detection to assist in training a Deep Q-Network (DQN). Doukhi et al. [36] developed an end-to-end mapless navigation system using 2D Lidar and depth images, applicable in both unknown indoor and outdoor environments. The architecture comprises a collision-awareness module (CAM) and a collision-free control policy module (CFCPM). The CAM processes and fuses sensory data to generate an observation, which is then passed to the CFCPM. In the CFCPM, a DQN algorithm with a CNN policy is employed to determine the optimal collision-free policy by selecting the best action from right, left, and forward movements. The authors in [26] proposed an indoor navigation system utilizing the DDPG algorithm. Their approach takes inputs such as a depth map, the drone’s current position, and the Euclidean distance to its destination. The reward function incorporates two margins. In the soft-margin area, a linear function gradually penalizes the drone as it approaches an obstacle. On the other hand, the hard-margin area uses a reciprocal function that rapidly increases the penalty to push the drone away from it. The research conducted by Li et al. [28] devised a UAV navigation system for a 3D environment using DDPG and TD3 algorithms. The observation space includes the UAV’s 3D orientation (position, velocity), environmental data from depth images, and information from 12 distance sensors. To ensure safe navigation across environments with varying levels of obstacles, the authors trained another deep learning model using human labeling to select appropriate reward schemes.

To capture temporal dependencies, researchers have applied various techniques such as recurrent neural networks (RNNs) and attention-based methods. In a study by Fu et al. [12], a memory-enhanced deep Q-network (DQN) algorithm was introduced for navigation, using visual inputs and kinetic data such as orientation, current position, and target location. Their proposed memory structure incorporates historical observations and actions, which are processed through an attention mechanism. Liu et al. [27] developed a UAV navigation system for indoor and narrow corridor environments, integrating hierarchical learning, recurrent neural networks, and the data-regularized Q (DrQ) algorithm. This system demonstrated improved handling of long input sequences and better exploration of temporal dependencies through temporal attention. While reinforcement learning (RL) has been successfully applied to navigation tasks in recent years, manually tuning hyperparameters, including reward functions, often leads to suboptimal solutions [37]. In multi-objective scenarios, determining the coefficients for reward functions involves significant trial and error, heavily relying on the experience of the researchers [38].

Due to the inability of monocular sensors to perceive depth information, researchers use various depth estimation techniques. Singla et al. [39] proposed a deep recurrent Q network with Long Short-Term Memory (LSTM) and temporal attention for obstacle avoidance in cluttered and unfamiliar indoor environments. The model takes monocular RGB images as input, which are transformed into depth maps using a conditional generative adversarial network (cGAN). The proposed approach by Xue et al. [21] involves using depth camera images as input, which are preprocessed using a variational autoencoder (VAE). The processed images are then utilized by the delayed soft actor-critic algorithm to generate continuous control commands. Kim et al. [20] employed a Fully Connected Residual Network (FCRN) to estimate depth from an RGB image, generating a corresponding depth image. These depth images, along with the RGB inputs, serve as inputs for the D3QN algorithm, which generates control commands. Although these approaches for obstacle avoidance have demonstrated satisfactory performance in indoor environments with limited obstacle settings, their effectiveness in outdoor environments with varied and complex obstacles remains untested. This is because the trained depth estimator learns only the features of known obstacles. To overcome this challenge, it would be beneficial to explore pretrained depth estimator models that are trained on various scenarios for depth estimation, such as DepthAnything [40], MiDaS [41] etc.

In recent years, large language models (LLMs) and vision-language models (VLMs) have proven to be highly effective for navigation tasks. Liu et al. [29] developed a system in which a UAV generates a series of actions based on natural language instructions, integrating both the instructions and various perception inputs like depth maps, RGB images, and pose data. This system is trained using flight trajectories collected with human support, with guidance from experienced annotators. Schumann et al. [30] created VELMA, which processes a sequence of prompts—including a task description, navigation instructions, and the current trajectory—to predict the next action through next-word prediction using an LLM. This action is executed in the environment, while landmarks identified from the instructions are verified for visibility in the panorama view at each step. The verbalizer combines this landmark data with potential intersection information to generate text observations, which are then added to the prompt sequence for ongoing action prediction until the agent reaches its target. In a similar approach, LM-Nav introduced in [42] handles raw observations and free-form textual instructions to create actionable plans using three pre-trained models: an LLM for landmark extraction, a VLM for grounding, and a visual navigation model (VNM) for execution. This enables LM-Nav to follow textual instructions in complex environments based solely on visual data, without needing fine-tuning. Rajvanshi et al. introduced sayNav for multi-object navigation tasks, where the UAV first constructs a 3D Scene Graph of various objects [31]. An LLM-based dynamic planner extracts a subgraph, converts it into text prompts, and inputs it into an LLM. Each planned step by the LLM is executed by Dagger, an imitation learning algorithm that produces control commands. In contrast to these approaches, our system operates independently of external instructions or additional environmental information.

## 3 Navigation task

The goal of the proposed VLM-Nav system is to autonomously guide a multirotor UAV from an initial position (*x*, *y*) to a target location (*x*_*dest*_, *y*_*dest*_). The UAV operates at a constant velocity of *v* and maintains starting altitude of *h*, adjusting its altitude only to avoid obstacles by moving vertically as needed.

### 3.1 Observation space

**Fig 2:**
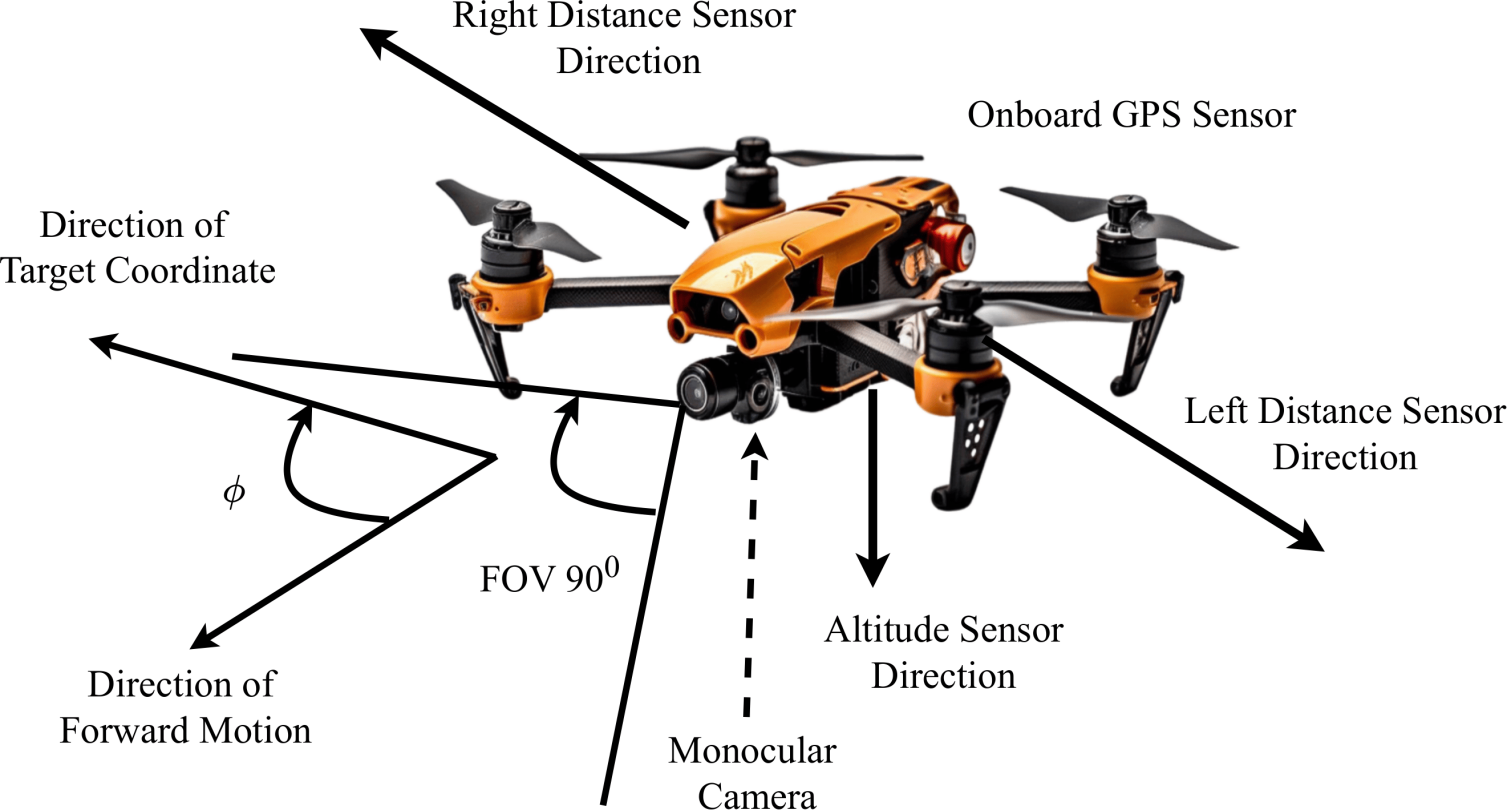
Navigation setup used in this study.

To navigate the UAV safely, in other words, to determine which action should be taken at each time step, the UAV uses state information from the environment. In this paper, the observation space, *S* can be expressed by equations 1 and 2. The drone captures RGB images *M* through a monocular camera. Then, a corresponding depth map for each image is generated, denoted by *D*. *L*_*dist*_ and *R*_*dist*_ represents the distances measured by the left and right distance sensors, respectively. ϕ is the angle between the drone’s forward direction and the target location. Fig 2 depicts the UAV setup for this study, highlighting different sensor placements.


D=DepthEstimation(M) (1)

$$S=\{D,L_{dist},R_{dist},\phi \}$$ (2)

### 3.2 Action space

The UAV observes its surroundings at every time step and generates low-level control commands. It is equipped with advanced autopilot systems that allow for straightforward control by specifying any particular position or velocity, with the control signals automatically generated by the autopilot system. We use Simpleflight, offered by Airsim [43], which aids our algorithm by facilitating seamless control between control commands and generated output. The action space encompasses five discrete actions, outlined as follows.


$$\begin{array}{cc} \text{Forward} & =\text{Go straight for 1s}\\ \text{Yaw Left} & =\text{Rotate counter clockwise by}\;25^{\circ }\\ \text{Yaw Right} & =\text{Rotate clockwise by}\;25^{\circ }\\ \text{Up} & =\text{Move upward for 1s}\\ \text{Down} & =\text{Move downward for 1s} \end{array}$$

### 3.3 Simulation environment

Simulation environments provide a secure, controlled space for testing and refining navigation algorithms, mitigating the risk of damaging expensive equipment or harming individuals or the drone. By simulating real-world scenarios and challenges, developers can evaluate the performance of navigation algorithms across various conditions, such as adverse weather, complex terrain, or unexpected obstacles. AirSim [43], a plugin for Unreal Engine (UE) developed by Microsoft, enables developers to implement their system for controlling multirotors in realistic 3-D environments. It is one of the most widely used platforms for autonomous vehicle research [21,26,44].

**Fig 3:**
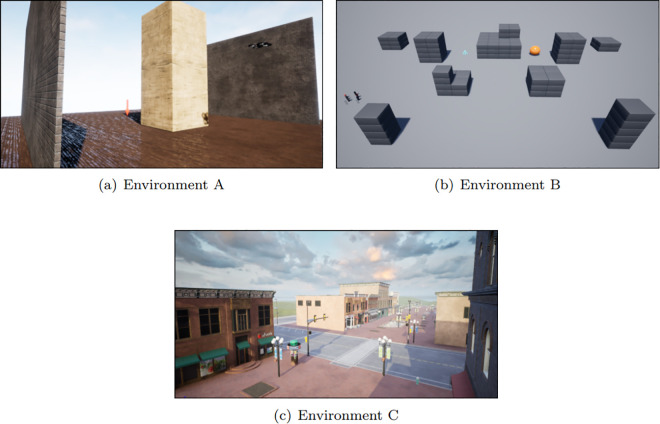
Our system is validated in the following three environments- (a) Simple environment with a single obstacle between two walls. This environment is used to train the navigator model. (b) The block environment is provided by AirSim. (c) Created using the Downtown West pack from the UE marketplace.

We have utilized three environments in UE for our research, as shown in Fig 3. Environment A, depicted in Fig 3, is a simple single-obstacle environment with two side walls, and its dimensions are 30m×30m . The width and height of the obstacle are randomly scaled by a factor of 0.5 to 5 during each navigation trajectory. Environment B, shown in Fig 3, is the Blocks environment provided by the AirSim package, featuring several obstacles of different shapes spaced throughout. It is comparatively large, with dimensions of approximately 220m×100m . Environment C, as shown in Fig 3, is made from the Downtown West pack, available in the UE marketplace. The pack contains assets of various objects in a city environment. Such as building, food cart, bench, rocks, posters, etc. We have made a customized environment using the pack to validate the UAV performance in realistic scenarios.

The objective for the UAV is to navigate from a given coordinate location to another while avoiding obstacles. At each episode, the spawn and target coordinates are generated randomly. Our approach enables the UAV to navigate from any starting point to the target location. Since the UAV will be flying at much higher altitudes in a practical scenario, we have ignored objects such as trees and poles. street lamp, etc.

## 4 Methodology

**Fig 4:**
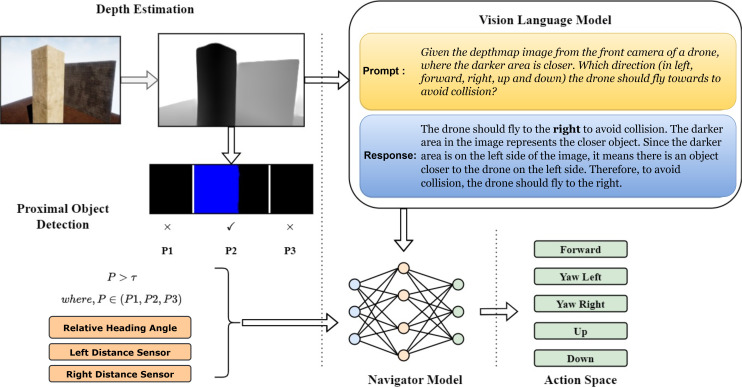
Overview of the proposed VLM-Nav method. (Left) First, RGB scene images are captured and converted into a depth map. (Top right) This depth map is then analyzed by the vision-language model (VLM), which provides the corresponding action response. (Bottom right) Lastly, the VLM’s feedback, along with the relative heading angle, left and right distance sensor measurements, and proximal object detection output, are sent to the navigation model.

This study develops a system for autonomous UAV navigation from a starting point to a target destination, using visual inputs to navigate around obstacles. The system functions in three phases, as shown in Fig 4. First, the UAV’s vision sensor captures monocular images, which are then converted into depth maps. These depth maps are analyzed by a Vision Language Model (VLM), which suggests actions for obstacle avoidance. In the final phase, a fully connected network processes data from depth map regions, distance sensor readings, heading angle, and VLM feedback to determine the UAV’s final action. Algorithm 1 presents the pseudocode for our proposed VLM-Nav approach.

**Algorithm 1: VLM-Nav Algorithm**

1: M← RGB scene

2: $L_{dist}\leftarrow$ Left distance sensor

3: $R_{dist}\leftarrow$ Right distance sensor

4: Prompt← VLM Prompt to avoid obstacle

5: τ← Threshold for proximal object detection

6: $\tau_{d}\leftarrow$ Threshold for distance sensors

7: $P_{current}\leftarrow$ Current Position

8: $P_{Target}\leftarrow$ Target Location

9: $d\leftarrow Distance(P_{current},P_{target})$

10: **while** d < 3m **do**

11:  ϕ← Relative heading angle

12:  D←DepthAnything(M)

13:  $D^{\prime }\leftarrow D>\tau$ // Cropped D of shape (m×n)

14:  $D^{\prime }\leftarrow Spaghetti(D^{\prime })$ // Connected Component

15:  $P1,P2,P3\leftarrow D^{\prime }$ //Three regions

16:  **for** P∈[P1,P2,P3] **do**

17:   n ← Number of connected groups ∈ P

18:   $P^{\prime }\leftarrow Bool(if\;2ptn>1)$

19:  **end for**

20:  $l\leftarrow Bool(L_{dist}>\tau_{d})$

21:  $r\leftarrow Bool(R_{dist}>\tau_{d})$

22:  F←VLM(D,Prompt)

23:  $States\leftarrow [P^{\prime },l,r,\phi ,F]$

24:  a←FCN(States) // navigator model

25:  step(a) //take action a

26: **end while**

### 4.1 Depth estimation

**Fig 5:**
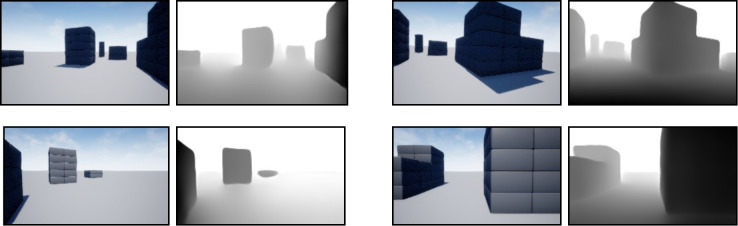
Examples of depth image estimation from Depth-Anything v2.

Effective navigation and obstacle avoidance require precise depth information. Since monocular images lacks spatial features, a real-time depth estimator is used in this study. While previous methods have employed various depth estimation techniques, such as FCRN and cGAN, they are generally trained on simulation engines. For this reason, a zero-shot depth estimation method is vital, allowing it to work with any images. DepthAnything [40] is a pre-trained, zero-shot depth estimation model designed to improve generalization using a dataset of 62 million automatically annotated, unlabeled images. A teacher model, initially trained on 1.5 million labeled images, generates pseudo-labels for the unlabeled data. The model follows an encoder-decoder architecture, with ViT-L (Vision Transformer large) as the encoder and DPT as the decoder. The combination of labeled and pseudo-labeled images is used to train a student model. To enhance the teacher model’s depth prediction, various perturbations, such as color distortions and Gaussian blurring, are applied, along with the integration of high-level semantic features. DepthAnything achieves superior zero-shot accuracy compared to models like MiDaS [41]. In DepthAnything V2 [45], manual labeling is replaced by more precise synthetic images from LiDAR and stereo sensors. The teacher is trained on these synthetic images, and to address distribution shifts and scene limitations between synthetic and real data, the model is jointly trained on pseudo-labeled real images, thereby enhancing its robustness and generalization to unseen data. In our work, real-scene images of size 144 × 256 captured by the UAV are processed with DepthAnything V2, with the outputs scaled to (0–255) and converted to single-channel images of the same shape. DepthAnything provides depth maps where the closer the distance, the larger the pixel value. To prepare the VLM for feeding, we first invert the depth maps. An example of a depth map generated by this module is shown in Fig 5.

### 4.2 Vision language model

With successful applications of vision language such as image captioning, segmentation, object detection, we are particularly interested in the visual question answering (VQA) task. VLMs such as Florence 2, PaliGemma, CogVLM are some of the popular options. However this algorithms mainly focus on vision task, and often offer limited language capability. In this work, we have utilized two language models with vision capabilities. One is GPT-4o (omni) model, a variant of generative pretrained transformer (GPT) architecture and is developed by OpenAI. GPT-4o stands out with improvements like larger context windows, faster processing, and efficient tokenization, excelling in text, audio, video, and image tasks [46]. Comparisons with top language models show GPT-4o’s superiority in performance metrics like throughput and response time.

**Fig 6:**
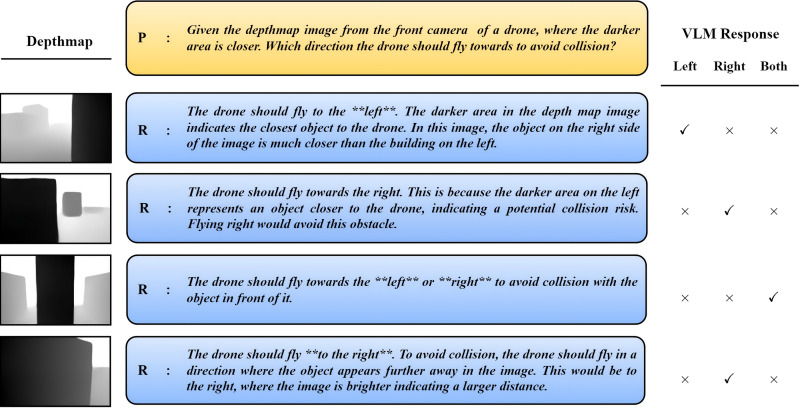
(left) The depthmap is estimated from the RGB scene, which is then normalized and rescaled into (0–255). (middle) The depthmap is sent to VLM along with the preset Prompt (P). (right) Based on VLM feedback (R), the suggested direction to avoid the obstacle is extracted using keyword search.

Another model is Gemini-1.5-flash model from the Gemini family developed by Google DeepMind [47]. Similar to the GPT-4o, this model can process multimodal data such as images, text, and videos, integrating advanced reasoning, planning, and memory capabilities making it highly versatile for complex real-world tasks [5]. This model is developed using Transformer Decoder architecture and is designed for more sophisticated problems across domains, offering superior performance in multimodal and complex reasoning tasks. Both models provides robust API support, making them suitable for integration into diverse applications without relying on large memory spacce, which is ideal for small UAV. Fig 6 illustrates how VLM is employed in VLM-Nav. The estimated depth map from the scene images is first normalized, rescaled, inverted, and then sent to the Gemini-1.5-flash model via an API key. The input to the VLM consists of a depth map and a predefined prompt that asks the UAV to take a direction to avoid colliding with any obstacles. The model provides the desired direction along with a detailed explanation for its response. Subsequently, directions such as left, right, or either direction are extracted using a keyword search, as shown on the left side of the figure. The incorporation of VLM significantly enhances generalization without requiring obstacle detection or semantic segmentation, which were used in previous navigation systems.

### 4.3 Proximal object detection (POD)

**Fig 7:**
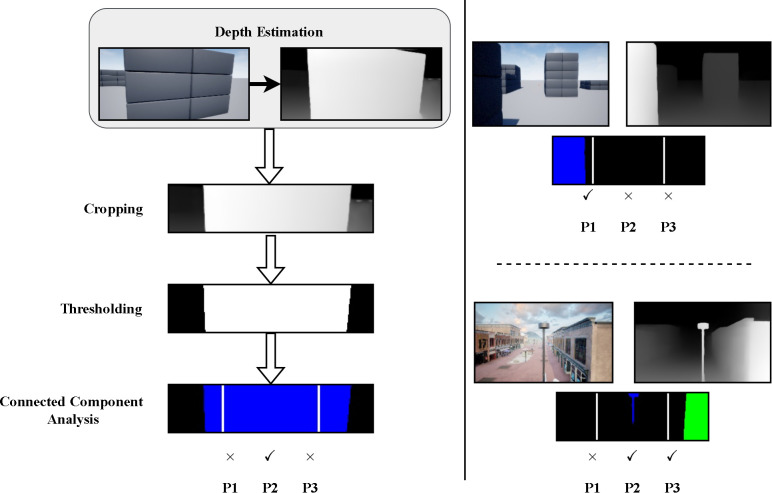
(Left) The process of proximal object detection in VLM-Nav: First, the depthmap is cropped, and the pixel values are binarized using a threshold τ, followed by connected component analysis. Finally, the output indicates whether any connected groups exist within the three defined regions. (Right) An example of the process with two scenarios.

We analyze the generated depth map as illustrated in Fig 7 to evaluate the distance to any obstacles that may exist in the front view. First, the depth map is cropped to a shape of (m×n). This cropping is especially necessary during low-altitude flight to exclude the ground and focus solely on the obstacles ahead. Next, binary thresholding is applied with a threshold value of τ, which helps ignore any obstacles beyond a certain distance. Afterward, connected component analysis is performed using the spaghetti technique [48], an efficient algorithm for identifying and labeling distinct connected components (i.e., clusters) in an image or grid. The algorithm is named after its approach of traversing a pixel grid in a manner akin to strands of spaghetti. First, the pixel grid is scanned row by row, from left to right and top to bottom. Labeling is then done by following the labels of neighboring pixels. In 8-connectivity, diagonal pixels are also considered. Only connected groups of more than 100 pixels are retained. We then divide the image into three sections (*P*1, *P*2, *P*3). The VLM (Gemini) cannot determine the distance to an obstacle based solely on the depth map. In fact, the depth map produced by DepthAnything does not provide true distances. These three sections serve two main purposes: first, they provide the UAV with a sense of obstacle depth. Secondly, they help reduce the number of API calls. We only send a request to the Gemini model for feedback if we detect any connected component in the P2 region, which is directly in front of the UAV. This significantly speeds up our system by avoiding request delays. Regions ‘P1’ and ‘P3’ assist the UAV in determining whether it is safe to turn, providing crucial information in such scenarios. The dimensions of the sections are carefully configured.

### 4.4 Navigator

**Fig 8:**
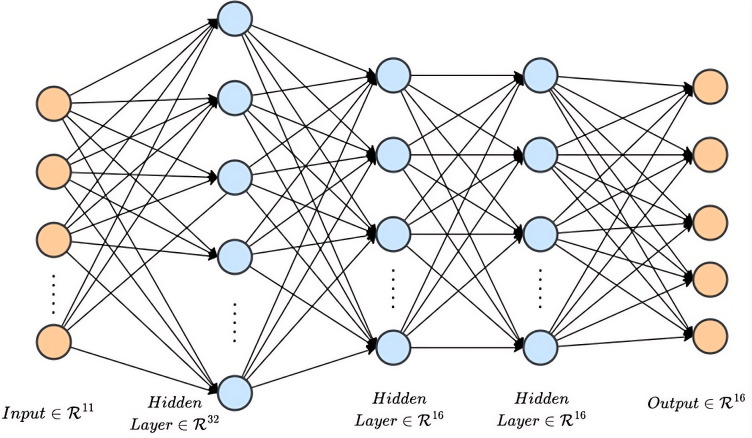
Navigator model architecture.

The navigator model in Fig 4 is a fully connected network (FCN) designed to mimic human-controlled drone flight. The RGB stream is converted to a depth map using DepthAnything V2, and then POD module detects the presence of any frontal obstacle using threshold value τ. Distance measurements from the left and right distance sensors are also collected. These distance values are compared against another threshold $\tau_{d}$. The purpose of this threshold is to maintain a minimum distance from an obstacle on the left or right while the UAV is flying beside it. It also aids the navigation task when rotating in a corner. The angle ϕ, which is the angle between the direction of the drone’s forward motion and the target location, is also calculated. We also incorporate feedback from the VLM module. This module provides a boolean array indicating the advised directions (Left, Right, Forward, Up, or Down). The array is generated through keyword searches in the VLM’s output text. In cases where an obstacle is directly ahead and the VLM suggests both left and right as optimal maneuvers as shown in Fig 6, both directions are set to True. Ultimately, the all these parameters are sent to an FCN, Which converts the multi-dimensional input into five discrete navigational actions (left, right, forward, up and down). The input parameters of the navigator model are provided in Table 3 in details. Fig 8 illustrates the model architecture.

**Table 3:** Details of input parameters for the navigator model.

Parameter	Type	Shape
Output from POD, $\left(P_{1}<\tau ?,P_{2}<\tau ?,P_{3}<\tau ?\right)$	(Bool, Bool, Bool)	(3,)
Relative heading angle, ϕ	Float	(1,)
Left distance sensor, $\left(L_{\text{dist}}<\tau_{d}?\right)$	Bool	(1,)
Right distance sensor, $\left(L_{\text{dist}}<\tau_{d}?\right)$	Bool	(1,)
VLM feedback (action to avoid collision)	(Bool, Bool, …, Bool)	(5,)
**Total Input Shape**	–	(11,)

To train the navigator model, we use the simplified environment A in Fig 3. A human annotator fly the UAV at each timestep while getting feedback from the input paramters. 10,000 steps of these parameters and the corresponding action taken by the human pilot is recorded as flight trajectories. These are then used to train the navigator model. The human annotators are instructed to maintain the following rules while selecting an action.

- Maintain a relative heading angle ϕ close to 0 during flight. Only initiate obstacle avoidance maneuvers when the POD module detects an obstacle ahead.
- To avoid obstacles, follow the direction provided by the VLM, unless it issues repetitive commands (e.g., alternating between left, right, and left) or generates incorrect directions.

Since the model does not rely on any images or location elements, it is completely map-independent and can be easily applied to other environments. Table 4 shows the hyperparameters of the navigator model.

**Table 4:** Hyperparameters of the Navigator FCN model.

Hyperparameter	Value
No. of hidden layers	3
No. of hidden Units / layer	32, 16, 16
Activation Function	ReLU
Loss Function	Cross-entropy
Optimizer	Adam
Batch Size	32

## 5 Result and discussion

The objective of this study is to develop an autonomous navigation system that enables a UAV to navigate from a randomly chosen spawn coordinate to a target coordinate while maintaining a fixed velocity and flight altitude. Different configuration settings for our approach are provided in Table 5.

**Table 5:** VLM-Nav Configurations.

Experiment Settings	Value
Scene dimension	(144 × 256)
Depthmap dimension	(144 × 256)
Velocity	3 meter/second
Initial altitude	10 meter
Yaw rate	25deg
τ	8
$\tau_{d}$	4
Connected component algorithm	Spaghetti
Minimum pixels in connected group	100
Command duration	1 second
Input token	137
Maximum Output token	300

### 5.1 Experimental setup

All experiments were carried out using the AirSim simulation plugin integrated with Unreal Engine version 4.27. The hardware platform for running the simulations was equipped with an Nvidia GeForce MX550 GPU and 16 GB of memory. This configuration ensured smooth rendering of the 3D environments and real-time data processing. The GPU’s high computational power was critical for handling the intensive tasks related to model training, inference and simulation in real-time.

### 5.2 Quantitative analysis

The initial stage of VLM-Nav entails creating a depth map from the UAV’s front-facing camera. We evaluated four algorithms for this task: MiDaS (based on DPT and Swin V2 models) and DepthAnything (versions V1 and V2). MiDaS offers various models depending on the underlying backbone architecture. We selected the DPT and Swin V2 large based models for experiments, as they offer an optimal balance between speed and accuracy. Table 6 shows a comparison of these methods. We gathered 10,000 scene images and corresponding ground truth depth maps from Unreal Engine in environments B and C. The ground-truth pixel values were generated by projecting rays from the camera to obstacles in Unreal Engine. These ground-truth values were then compared with the estimated depth maps, averaging the results across the 10,000 images using the following metrics.

**Table 6:** Performance comparison of depth estimation algorithms.

Method	Env.	$\delta_{1}↑$	$\delta_{2}↑$	$\delta_{3}↑$	AbsRel ↓	SSim Index ↑	Inference Time(ms) ↓	Par. (M) ↓
MiDaS (DPT)	B	0.5813	0.7807	0.9029	1.4887	0.5956	62.07	344
	C	0.6677	0.8615	0.6617	1.2940	0.5283	66.64	
MiDaS (Swin V2)	B	0.4232	0.6452	0.7586	0.8043	0.3034	1044.9	213
	C	0.6160	0.8629	0.7632	0.6246	0.5490	1027.2	
DepthAnything-V1	B	0.4196	0.6578	0.6783	0.4722	0.8507	23.77	24.8
	C	0.5801	0.7846	0.9414	0.1498	0.8899	21.07	
DepthAnything-V2	B	0.4590	0.7110	0.7930	0.3904	0.8901	22.93	24.8
	C	0.5125	0.7311	0.8963	0.4723	0.7439	24.94

1. Delta (δ): It computes the ratio of depth predictions $\hat{d}$ that fall within a certain threshold $\delta_{t}$ of the true depth *d* as shown in Eq. 3. Typically, multiple thresholds (e.g., $\delta_{1}<1.25$, $\delta_{2}<1.25^{2}$, $\delta_{3}<1.25^{3}$) are used to assess different levels of accuracy. Higher delta values indicate better performance.
  $$\delta =\max\left(\frac{\hat{d}}{d},\frac{d}{\hat{d}}\right)<\delta_{t}$$ (3)
2. Absolute Relative Error (AbsRel): This metric calculates the absolute difference between predicted ${\hat{d}}_{i}$ and true depth values *d*_*i*_, normalized by the true depth as shown in Eq. 4. It shows how far the predictions deviate from the ground truth on average, scaled by the actual depth values. Lower AbsRel scores indicate better performance.
  $$\text{AbsRel}=\frac{1}{N}\sum_{i=1}^{N}\frac{\left|{\hat{d}}_{i}-d_{i}\right|}{d_{i}}$$ (4)
3. Structural Similarity Index (SSIM): SSIM measures the similarity between the predicted and true depth maps in terms of structure, luminance, and contrast, taking into account spatial relationships. It evaluates the perceived quality of the depth estimation and measured using Eq. 5. SSIM ranges from −1–1, where values closer to 1 indicate higher similarity.
  $$\text{SSIM}(\hat{d},d)=\frac{\left(2\mu_{\hat{d}}\mu_{d}+C_{1})(2\sigma_{\hat{d}d}+C_{2}\right)}{\left(\overset{2}{\underset{\hat{d}}{\mu }}+\overset{2}{\underset{d}{\mu }}+C_{1})(\overset{2}{\underset{\hat{d}}{\sigma }}+\overset{2}{\underset{d}{\sigma }}+C_{2}\right)}$$ (5)
  Here, $\mu_{\hat{d}}$ and $\mu_{d}$ are the means of the predicted and ground truth maps, $\overset{2}{\underset{\hat{d}}{\sigma }}$ and $\overset{2}{\underset{d}{\sigma }}$ are the variances, $\sigma_{\hat{d}d}$ is the covariance between the two images, *C*_1_ and *C*_2_ are small constants to avoid division by zero.
4. Inference time: It measures the time required for each method to estimate depthmap from a single scene image and indicates how fast the algorithm is.
5. Parameters (Par.): The number of trained weights in the model and presented in milions. Higher number requires more computational resources.

Both DepthAnything V1 and V2 significantly outperform the other techniques, demonstrating superior performance. These models are particularly well-suited for deployment in UAV systems due to their low parameter count and fast inference speed. Additionally, they offer higher structural similarity compared to the other models. While V1 and V2 are quite similar, V2 shows a slight edge in performance in both environments B and C.

**Fig 9:**
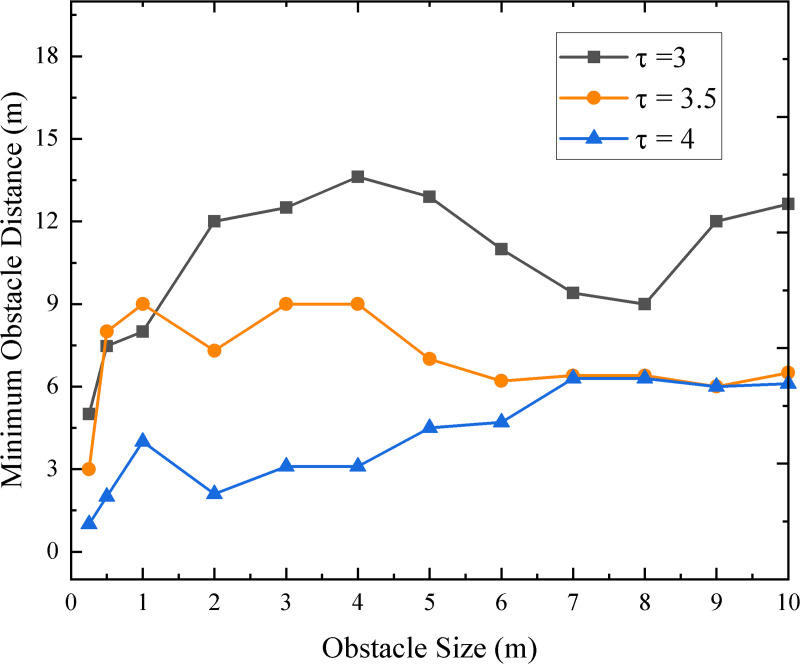
Minimum distance to obstacle detection based on different value of thresholds.

The POD module in our method estimates whether any obstacles within a certain distance, defined by τ, exist as connected groups in three regions of the depth map. These three regions capture only a small portion of the depth map and provide limited depth information from the UAV’s front perspective. In contrast, the VLM analyzes the entire depth map to identify obstacle locations and determine the appropriate direction to avoid them. A higher threshold value allows early detection, while a lower value requires the UAV to get closer. However, the POD module largely depends on the accuracy of the estimated depth map. DepthAnything v2 struggles to generate accurate depth values for distant small obstacles. As the obstacle approaches during navigation, the depth map improves, allowing the POD module to detect it. Fig 9 shows how our system detects cubes of different sizes, from 25 cm to 10 m, at varying distances depending on τ. Smaller obstacles require the UAV to be closer for detection, while larger obstacles are detected from farther away.

The navigator module in our method is an FCN that uses feedback from a VLM, distance sensor measurements, object detection from the POD module, and the heading angle to select the optimal action from five discrete options. The model is trained in a multiclass classification framework using a dataset gathered from human-operated flights in environment A. To evaluate the model’s performance, we collected human-annotated trajectories in two different environments, B and C. For each discrete action class, we measured true positives (TP), true negatives (TN), false positives (FP), and false negatives (FN) using a one-vs-all approach (treating each class as positive and all others as negative), and then averaged these values across classes. TP and TN represent how accurately the model predicted the correct actions relative to human annotations. FP and FN capture the instances of incorrect predictions, where the model either predicted a wrong action (FP) or missed the correct one (FN). These metrics were then used to compute precision, recall, and F1 score, which we briefly discuss with respect to the navigation task as follows:

- Precision: It refers to the number of correct actions out of predictions and calculated using Eq. 6. Higher value means the model often predicts correct actions.
  $$Precision=\frac{TP}{TP+FP}$$ (6)
- Recall: It indicates out of all the times the model should have predicted a specific action, how often did it actually predict it. High recall ensures that the model catches most of the correct actions.
  $$Recall=\frac{TP}{TP+FN}$$ (7)
- F1 Score: As shown in Eq. 8, it is the harmonic mean of precision and recall. It provides a single measure that balances both precision and recall.
  $$F1Score=2\times \frac{Precision\times Recall}{Precision+Recall}$$ (8)

Table 7 presents the performance of the navigator module across all environments. The results indicate that both the Gemini and GPT-4o models achieve high accuracy, with F1 scores around 90% in previously unseen environments (B & C). This demonstrates the strong generalization capability of our approach. While both models perform well, the Gemini Flash model shows a slight edge in performance compared to the GPT-4o model, indicating its enhanced adaptability in navigating new environments. The overall navigation performance is then assessed by averaging the results of 100 flight trajectories and is presented in Table 8, using three key metrics:

**Table 7:** Performance of navigator module.

VLM	Environment	Precision ↑	Recall ↑	F1-Score ↑
**Gemini**	A	**0.9978**	**0.9889**	**0.9933**
	B	**0.9149**	0.9034	**0.9070**
	C	**0.9129**	**0.8961**	**0.9026**
**GPT 4**	A	0.9921	0.9875	0.9899
	B	0.8932	**0.9046**	0.8988
	C	0.9089	0.8742	0.8912

**Table 8:** Overall navigation performance of VLM-Nav.

VLM	Depth Estimator	Environment	TCR ↑	CR ↓	TLR ↑
**GPT-4**	DepthAnything-V1	A	1.00	0.00	0.98
		B	1.00	0.00	0.98
		C	0.98	0.06	0.93
	DepthAnything-V2	A	1.00	0.00	0.99
		B	1.00	0.00	0.99
		C	1.00	0.02	0.93
**Gemini**	DepthAnything-V1	A	1.00	0.00	0.99
		B	1.00	0.01	0.98
		C	1.00	0.05	0.92
	DepthAnything-V2	A	1.00	0.00	0.99
		B	1.00	0.00	0.99
		C	0.98	0.03	0.94

1. Task Completion Rate (TCR): The navigation task is deemed successful if the UAV reaches within 3 meters of the target location; otherwise, it is considered a failure. TCR represents the percentage of successful flight trajectories.
2. Collision Rate (CR): This measures the percentage of times the UAV collides with an obstacle. It is calculated by summing collisions across the entire trajectory and converting to percentage with respect to distance travelled during flight as shown in Eq. 9.
  $$CR=\frac{Number\;of\;Collisions}{Total\;Distance\;(m)}$$ (9)
3. Trajectory Length Ratio (TLR): This metric measures the ratio of trajectory length of the human-operated UAV (*L*_*H*_) and the proposed system $\left(L_{VLM-Nav}\right)$ as shown in Eq. 10. It indicates whether the UAV follows a path close to the shortest route to the target and mimics human decision making. Values close to 1 indicates better formance.
  $$TLR=\frac{\min(L_{H},L_{VLM-Nav})}{\max(L_{H},L_{VLM-Nav})}$$ (10)

From the table, it is evident that despite being trained in the simple environment, the navigator model achieves an almost perfect TCR in both the blocks and downtown west environments. Additionally, the system maintains a very low collision rate for static obstacle, though collisions tend to increase when encountering smaller obstacles or when the depth map estimation is inaccurate. For this reason we have also observed higher collision rate with DepthAnything V1 model compared to V2. The low TLR values suggest that the system closely follows the human-guided navigation path. The inference time of VLM-Nav is shown in Table 9. While components like depth estimation and POD have a minimal impact on the total time, the VLM module consumes the most time, approximately 1 second, primarily due to the delay from the API call. Despite this, the VLM significantly improves system performance, making it domain-invariant and reliable in unseen environments. To address this, we use POD, which skips the API call when no frontal obstacle is detected, reducing the total runtime to just 108 milliseconds.

**Table 9:** Inference time of VLM-Nav.

Module	Time (mS)
State collection	25
Depth Estimation	24.94
Proximal Object Detection	1
VLM Feedback	1000
Navigator Model	1
Total	51.94 / 1052

### 5.3 Qualitative analysis

**Fig 10:**
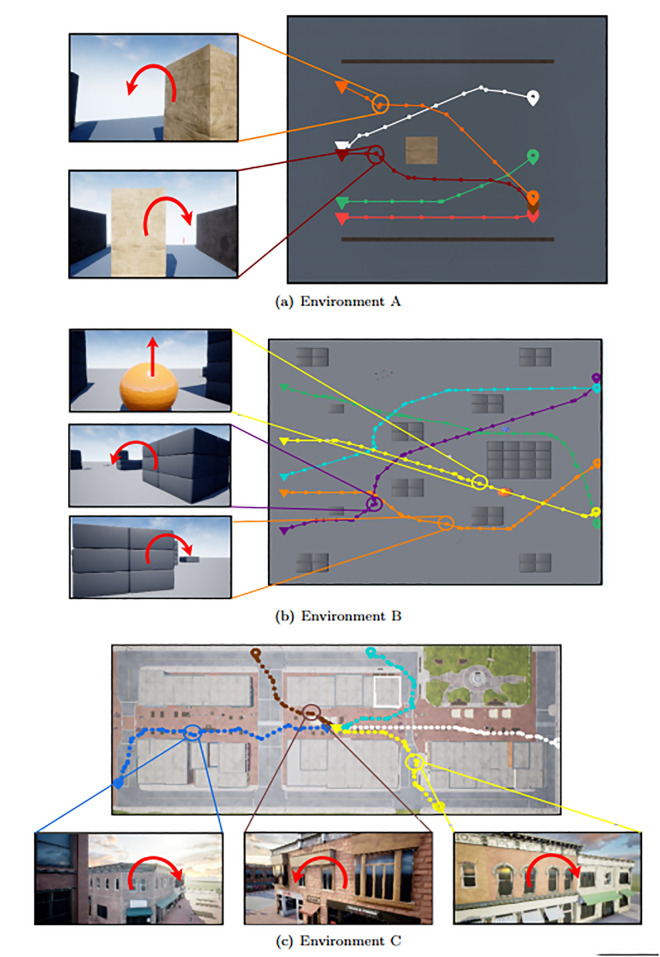
Five example flight paths generated by VLM-Nav in three different environments are presented. The starting and target coordinates are marked by (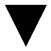) and (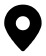) symbols, respectively. Selected points (indicated by circles) along the flight paths are shown from the UAV’s front camera perspective. At these points, the UAV’s movement direction, taken to avoid obstacles, is depicted with red arrow symbols (e.g., 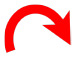 for yaw right, 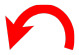 for yaw left, and 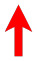 to go upward).

**Fig 11:**
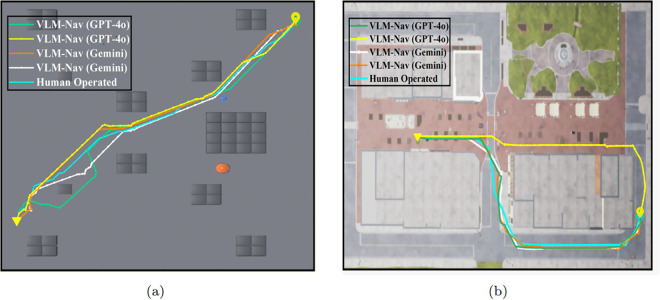
The flight path comparison between VLM-Nav and human-controlled flight in (a): Blocks and (b) Downtown West environment. The starting and target location is shown using color (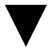) and (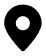) markers.

After training the navigator model, the approach is validated in environments B and C within Unreal Engine. Fig 10 shows a top view of the three environments (simple, blocks, and downtown west). The flight paths of ten consecutive flights following the proposed model are illustrated with a colored plot, where each color represents a different episode. In the simple and block environments, the spawn and target coordinates are randomly selected from fixed start and end zones, which can be seen at the top and bottom of Fig 10A and Fig 10B, respectively. However, in the downtown west environment, the starting zone is in the middle of the map, and the target coordinate is selected around the perimeter edges of the map. The proposed method successfully navigates to the target location without colliding with any obstacles while keeping the flight path as short as possible. The navigator model attempts to replicate human flight patterns based on inputs from other components of our system. Fig 11 illustrates three flight paths generated by VLM-Nav after training the navigator model, which is then compared to a human-guided flight from the same starting point to the target location. It is evident that the flight paths generated by our system closely resemble the human-controlled flights in both the blocks and downtown west environments.

An additional observation we made was that when complex instructions are given to VLM, it produces inconsistent output. We have implemented faster, simpler prompts for each API call to reduce the impact of these problems and ensure more reliable results. In addition, it is essential to keep in mind that not all VLMs are capable of managing depth maps. Therefore, selecting a VLM that supports depth maps is essential for applications that rely on depth information for accurate decision-making and action execution.

### 5.4 Comparative analysis

In recent studies, many researchers focus on qualitative analysis without directly reporting key metrics such as test time and computational cost. The study in [49] addresses the challenge of UAVs struggling to generalize in unseen environments. Traditional DRL models often rely on non-causal features, which are not directly related to the task, leading to performance degradation in new settings. The paper introduces Causal Feature Selection (CFS), which prioritizes causal features that directly impact navigation and obstacle avoidance. By filtering out non-causal features, the UAV’s model becomes more adaptable, improving the Success Rate (SR) from 60% to 85% in unseen scenarios. However, the method only works with simple geometric obstacles and has low computational cost. In [50], Wei et al. proposed an approach using Dual-Transformer Encoders within PPO to improve multi-UAV collaboration.

This method increased transferability by 30% and obstacle avoidance success by 20% (from 70% to 90%) in dynamic, complex environments. In contrast, Javaid et al. [51] presented a monocular vision-based obstacle-avoidance system for UAVs that uses depth-estimation models to detect obstacles. This method achieves high SR across varying light conditions (83.33% in high light, 100% in moderate light), though it struggles in low-light and dynamic environments. It is cost-effective but has limitations in extreme lighting. LMNav, developed by Shah et al. [42], enables UAVs to follow natural language instructions using pre-trained models such as GPT-3, CLIP, and ViNG. Unlike traditional models, it doesn’t require fine-tuning and is more generalizable, but it is computationally expensive and struggles with visual grounding and landmark recognition in complex settings [52]. In comparison, VLM-Nav excels at generalizing to unseen environments, with a high task completion rate. It is a lightweight, fast method that requires no extensive pretraining, making it more efficient than models like LMNav, which rely on large, computationally intensive pre-trained models. Table 10 highlights how VLM-Nav outperforms these methods, offering a more efficient and adaptable solution for UAV navigation.

**Table 10:** Comparison of our approach with recent studies in UAV navigation.

Author	Computing Cost	Inference Time	Result	Pros	Cons
Zhuang *et al.* [49]	Low–moderate	Low–moderate	85% SR in unseen env.	• Generalizes better by filtering out irrelevant features (background texture). • Improved accuracy on unseen environments.	• May require significant preprocessing. • Implemented with simple geometric obstacles.
Wei *et al.* [50]	Moderate–high	High	90% SR	• Improves generalization of multi-UAV systems in unseen environments. • Increased transferability and obstacle avoidance success rate.	• Computationally intensive due to the use of dual transformer encoders.
Javaid *et al.* [51]	High	Approx. 3s	80–100% SR (lighting-dependent)	• Cost-effective and lightweight. • Capable of operating in low light conditions up to a certain threshold.	• May experience false positives/accidents in challenging conditions (e.g., reflective surfaces). • Requires many processing steps which adds delay.
Shah *et al.* [42]	High	High	85% SR	• Capable of generalizing to new environments without requiring fine-tuning. • Suitable for practical robotic navigation with long-horizon tasks (over 100 meters).	• Depends heavily on landmark-based navigation. • Requires human instructions throughout the navigation step.
Proposed VLM-Nav	Low	Low (52ms without & 1s with obstacles)	98% Success Rate	• Strong generalization in unseen maps. • Real-time navigation and obstacle avoidance. • Doesn’t require extensive training.	• Works only with static obstacles.

**Fig 12:**
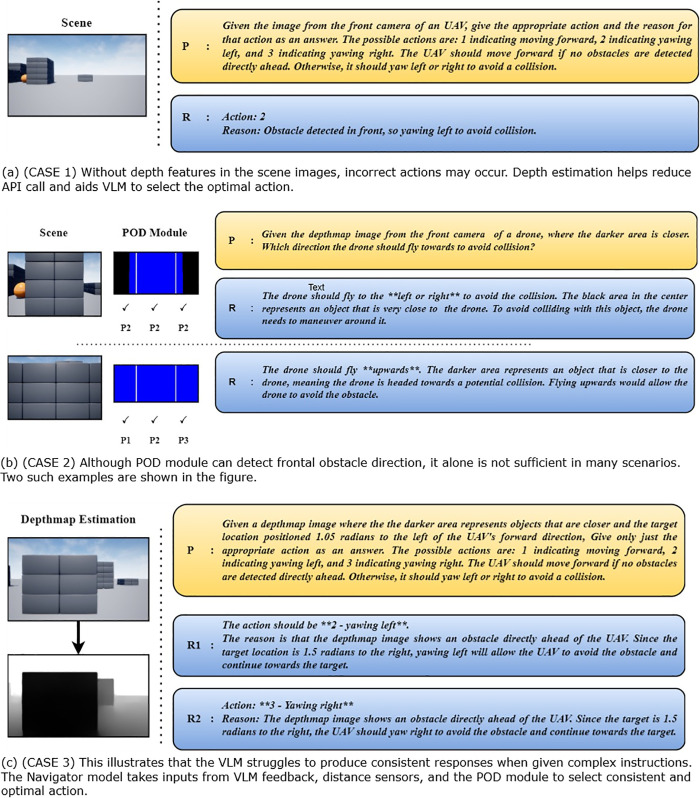
Ablation case studies.

### 5.5 Ablation study

This section explains how each component contributes to the navigation system as a whole. Three cases, illustrated in Fig 12A-Fig 12C are presented to demonstrate this. While the VLM module has strong reasoning abilities for detecting and avoiding obstacles, it lacks spatial awareness, making depth map estimation necessary. Case 1 in Fig 12A, for example, the VLM is given an RGB scene image and asked to navigate by choosing one of three actions. As shown, despite the obstacle being far away, the UAV still chooses to rotate because it cannot determine the object’s distance. Without depth information, detecting nearby obstacles in POD module is also not feasible.

In our system, the goal of POD module is to determine whether there is any obstacle closeby in front of the UAV. The obstacle is represented by connected components and the depthmap is divided into three regions. However, relying solely on the proximal object detection module for navigation isn’t sufficient. Often times, in situations where the drone is directly facing the obstacle at a close distance as shown in Case 2 in Fig 12B, decision-making becomes difficult. In such cases, the VLM analyzes the entire depth map and determines which direction is best for avoiding collisions. Although VLMs show great potential in various applications, their performance can be inconsistent, especially with complex prompts. This complexity often leads to variability in the VLMs’ results, affecting the reliability of their outputs. The inconsistency is more evident when VLMs must follow detailed instructions. Case 3 in Fig 12C, for example, the VLM is given an image and angle ϕ to generate a navigation action. Despite the target being 1.5 radians to the right and the right side being obstacle-free, the VLM produces two different responses. Therefore, simpler prompts, as used in our system, yield more consistent and meaningful results. Then they are supported by the POD module and navigator model.

## 6 Conclusion

This research presents VLM-Nav, a cost-effective UAV navigation system that uses monocular vision and advanced vision-language models (VLM) for robust autonomous navigation. Using a single RGB camera with depth estimation eliminates the need for expensive sensors while maintaining high accuracy. Integrating state-of-the-art VLMs such as Gemini-flash-1.5 and GPT-4o improves decision making. The proposed approach is trained to mimic human controlled flight, which is then verified using the Airsim simulator in Unreal Engine. Using a zero-shot depth estimator and a VLM to avoid obstacles, this approach overcomes the limitation of generalization in unfamiliar environments highlighted in previous research. Future work will explore the deployment of lightweight, on-board vision–language models on edge computing platforms such as NVIDIA Jetson Orin to reduce latency and enable fully offline operation. Extending the navigation module to continuous control is expected to generate smoother and more energy-efficient trajectories, improving flight stability and performance. Further research could also investigate creating a specialized VLM for UAV navigation that functions without depth estimation and can interpret complex instructions for consistent results. Dynamic obstacle avoidance can also be explored with the help of VLM. For example, it might be possible to use the generated depthmap as input for models such as spatio-temporal graph neural network and considering multiple timesteps to predict the movement pattern of objects. The proposed method operates independently of the instructions of the human pilot, providing valuable information and directions for integrating VLMs into autonomous UAV systems for practical applications such as parcel delivery, surveillance, and disaster management.
